# Missed pulmonary tuberculosis: a cross sectional study in the general medical inpatient wards of a large referral hospital in Ethiopia

**DOI:** 10.1186/s12879-019-3716-x

**Published:** 2019-01-17

**Authors:** Dawit Assefa, Feleke Belachew, Getachew Wondimagegn, Eveline Klinkenberg

**Affiliations:** 1 USAID/Challenge TB, Addis Ababa, Ethiopia; 2 Oromia Regional Laboratory, Ministry of Health, Oromiya, Ethiopia; 3 KNCV Tuberculosis Foundation, The Hague, Netherlands; 4 Department of Global Health, Amsterdam Institute for Global Health and Development, Academic Medical Centre, Amsterdam, The Netherlands

**Keywords:** Pulmonary Tuberculosis, TB screening, Clinical practice, GeneXpert testing

## Abstract

**Background:**

Every year around 4 million people with tuberculosis (TB) are not detected. Thus may not get the medical care that they need and deserve from their respective health systems. Ethiopia is included in the 12 countries who contribute 75% of the globally estimated “missed” cases. This study assessed if there are missed Pulmonary TB (PTB) cases among inpatients of a large referral hospital in Ethiopia.

**Method:**

A cross sectional survey was conducted in the general medical wards of the large referral hospital from June to August 2015. Inpatients not diagnosed with TB were screened for TB symptoms and requested to submit a morning sputum sample for smear microscopy and molecular testing by GeneXpert MTB/RIF assay. The results of the symptom screening, smear and GeneXpert testing were analyzed as the main outcome characteristics for “missed” PTB cases.

**Result:**

Over the 3-month period, 300 inpatients were evaluated for TB. The patients median age was 38 years (IQR 26–51.5), 41% were female, median reported duration of sickness before admission was 30 days (IQR 14–240), and median body mass index (BMI) was 21.5 (IQR 20–22.67). HIV status was documented for 198/300 (66%) of patients, 37 (18.7%) were found to be HIV positive, with a median CD4 count of 176 (IQR 52–400). All 300 inpatients submitted a sputum sample and 10 (3.3%) were found to be GeneXpert MTB positive, with 4/10 also being smear positive. All GeneXpert positive inpatients reported having a cough of > 2 weeks duration. Eight had at least 3 common symptoms of TB (i.e. cough, fever, weight loss or night sweat). Co-morbidity with Diabetes Mellitus (DM) and HIV was found in 1/10 and 4/10 cases respectively.

**Conclusion:**

Bacteriological confirmed TB cases were found to have been “missed” amongst the general medical ward inpatients in the hospital. The identified TB cases all reported typical signs and symptoms of TB. Basic clinical practices were not being followed (i.e. history taking/documentation and requesting of appropriate laboratory tests) by the attending health care workers (HCWs) in the hospital. The index of suspicion for TB disease needs to improve and the use of more sensitive technologies, such as GeneXpert could assist the diagnosis of TB. However, the findings of the study need to be investigated in other hospital settings in Ethiopia.

## Introduction

Globally, about 4 million people with tuberculosis (TB) are missed each year by the health system. Of these missed cases 75% are in 12 countries and Ethiopia is one of them [1]. From the estimated 200,000 TB cases (all forms) occurring annually in Ethiopia, in 2015 only 137,960 (68%) were notified [2]. Hence about one-third of TB cases are missed or not notified in the country. Although the directly observed therapy, short-course strategy (DOTS) has been scaled up globally between 2000 and 2014, with 43 million lives saved, 1.5 million people still die every year from TB [1]. Most of these deaths reflect diagnosis that is either delayed, missed, or never attempted [1].

In most high TB burden countries, diagnostic delay among TB patients is common and long. Such delay may lead to more extensive disease, complications and increased chance of death [3–5]. Delayed TB diagnosis has also been observed in low TB burden countries such as in United States and Canada. For instance, among hospitalized patients, respiratory-related hospitalization or visits were found to be common until approximately 90 days before the TB diagnosis, primarily due to healthcare professionals not suspecting TB in patients with respiratory-related problems [6]. Failure to diagnose and treat TB early puts family members and the community at increased risk of TB infection and subsequent disease. In addition, the complex contact patterns between healthcare workers and patients potentiate the spread of TB disease in the healthcare setting, resulting in an increased occupational risk of TB infection and disease among health care workers (HCWs) and practicing medical students [7–10].

The 2014–2020 Ethiopia National TB Strategic Plan (NSP) emphasizes the need to improve access to quality TB, TB/HIV and multidrug-resistant TB (MDR-TB) services in order to curb transmission and reduce the disease burden through early detection and treatment of infectious TB cases [11]. The key strategies of the NSP for implementation are: (i) improving diagnostic capacity (e.g. scale up of new tools and technologies, such as the GeneXpert MTB/Rif assay); (ii) interventions that aim to reduce patient delay (e.g. community awareness); and (iii) targeted screening that does not rely on patient presentation (e.g. screening of contacts and high risk groups) [11, 12].

The aim of the current study is to assess if there are patients admitted in the general medical wards of a large referral hospital in Ethiopia with TB symptoms (cough ≥2 weeks duration) who have not received the required investigations (e.g. TB symptoms screen, sputum test). We hypothesize that pulmonary TB (PTB) cases are missed while being treated for other medical conditions. In addition, if we are able to find missed TB cases in the general medical wards, it would highlight the need for HCWs to strictly adhere to infection control (IC) practices as undiagnosed PTB cases could potentially be a source for nosocomial transmission of TB infection.

## Materials and methods

The Oromiya Region is one of the largest provinces of Ethiopia, with around one-third of the country’s total population. This study was conducted in one of the largest referral teaching hospitals of the Oromiya Region. The facility was purposefully selected because of its high workload and that it provides referral services for both urban and rural communities, with a catchment population of around 753,000. On average, the hospital notifies 150 to 200 TB cases (all forms) per quarter.

A cross-sectional survey was conducted in the male and female general medical wards of the hospital from 1 June to 30 August 2015 (i.e. over a 3 month period). All patients with a non-TB medical diagnosis who were admitted to the general medical wards, were interviewed and their medical records reviewed. A structured data capture form was used to collect demographic and clinical characteristics and information related to current or past episodes of TB. Patients with a diagnosis of any form of TB and patients who were referred for sputum testing for suspected TB disease, were excluded from the study analyses. All included patients were also requested to submit a morning sputum sample for smear microscopy and GeneXpert testing. These tests were carried out at the quality assured regional TB laboratory in Adama (Oromiya). Patients detected as a case of bacteriologically confirmed TB were registered at the hospital and put on anti-TB treatment as per the national guidelines.

### Operational definitions

Missed TB case: A patient admitted to a general medical ward of the hospital with a non-TB diagnosis, who was not suspected by the hospital staff of having TB disease, and who was diagnosed as having TB disease during the study.

Suspected TB case: A patient admitted to a general medical ward of the hospital with a non-TB diagnosis, who was not suspected of TB by the hospital staff, but who was found to have ‘cough of more than 2 weeks duration’ during the study.

Bacteriologically confirmed pulmonary TB case: A patient whose submitted sputum sample was positive either by smear microscopy and/or GeneXpert test.

Patient with drug-resistant TB: A patient found to be “MTB positive / Rifampicin Resistant” by GeneXpert testing.

### Data collection and analysis

Two trained nurses working in the general medical wards conducted the interviews and extracted the information from the patients’ medical records. They were regularly supervised by the principal investigator. Data were double entered, cleaned and validated, and then analyzed using SPSS version 7.1. Descriptive statistical analyses (frequency, medians and interquartile ranges [IQRs]) were carried out on the patients’ demographic and clinical characteristics and their laboratory results. Bivariate analysis was done to investigate the association between demographic and clinical characteristics, TB screening and laboratory results.

## Results

In the 3-month period, 327 patients with medical illnesses were admitted to the male and female general medical wards. Of the 327, 12 patients were admitted with a differential diagnosis including TB or with diagnosed TB (any form). Fifteen others were not able to submit sputum for various reasons. These 27 persons were excluded from further analysis. Thus, in total, the data capture form was filled and a morning sputum sample collected for microscopy and GeneXpert testing for 300 inpatients. The median age was 38 years (IQR 26–51.5) and 177 (59%) were male. A total of 160 (53%) had completed primary and secondary level of education, while 100 (36%) were illiterate and 32 (10.7%) had college diploma. With regards to income, 193 (64.3%) owned small private business, 86 (28.7%) had no income and the remaining 21(7%) were government employees (Table 1). A total of 28 (9.3%) of the patients said that they currently smoke cigarettes.

**Table 1:** Socio-demographic and clinical characteristics of the study participants (*n* = 300)

Characteristics	N (%)
Median age (IQR)	
38 (26–51.5)	
Sex	
Male	177 (59.0)
Female	123 (41.0)
Education level	
Cannot read and write	108 (36.0)
1-8th grade school	100 (33.3)
8 – 12th grade school	60 (20.0)
College	32 (10.7)
Income	
Employed (government or private)	213 (64.3)
Unemployed (no income)	87 (6.7)
Median body mass index/BMI (IQR)	
21.5 (20–22.6)	
HIV status	
Positive	37 (12.3%)
Negative	161 (53.7)
Not documented	102 (34.0)
Median CD4 count (IQR)	
176 (52–400)	
On antiretroviral treatment / ART	
Yes	27 (73.0)
No	10 (27.0)
Median duration of current sickness (days) (IQR)	
30 (14–240)	

The median duration of sickness for which the patient was hospitalized was 30 days (IQR 14–240) and the median body mass index (BMI) was 21.5 (IQR 20–22.67). HIV status was documented for 198/300 (66%) inpatients, of which 37/198 (18.7%) had a positive HIV test result. The median CD4 count for the 37 HIV positive patients was 176, and 27/37 (73%) were on anti-retroviral treatment (ART). Thirty eight (12.7%) were found to have diabetes mellitus, and 3 (1%) had a chronic respiratory problem (e.g. chronic bronchitis, asthma).

Before their current admission, 243 (81%) patients had visited other public health facilities, 48 (16%) went to private health clinics, 5 (1.6%) had consulted a traditional healer, with just 4 (1.3) reporting no prior visits to another health facility. In regards to TB related symptoms, 187 (62.3%) reported a cough of any duration, with 125 (66.8%) having a cough of two or more weeks duration i.e. were cases of presumptive TB. One hundred and forty (46.6%) patients had a history of fever, and 26 (8.7%) reported a history of contact with a known TB patient (Table 2).

**Table 2:** Common TB symptoms and GeneXpert positivity amongst screened inpatients (*n* = 300)

TB symptoms	Yes	GeneXpert positive	Prevalence (%)
Cough of any duration	187 (62.3%)	10	5.3
Cough ≥2wks	125 (41.7%)	10	8.0
Fever	140 (46.7%)	9	6.4
Night sweat	74 (24.7%)	4	5.4
Chest pain	98 (32.7%)	3	3.1
Contact history	26 (8.7%)	1	3.8

**Fig. 1:**
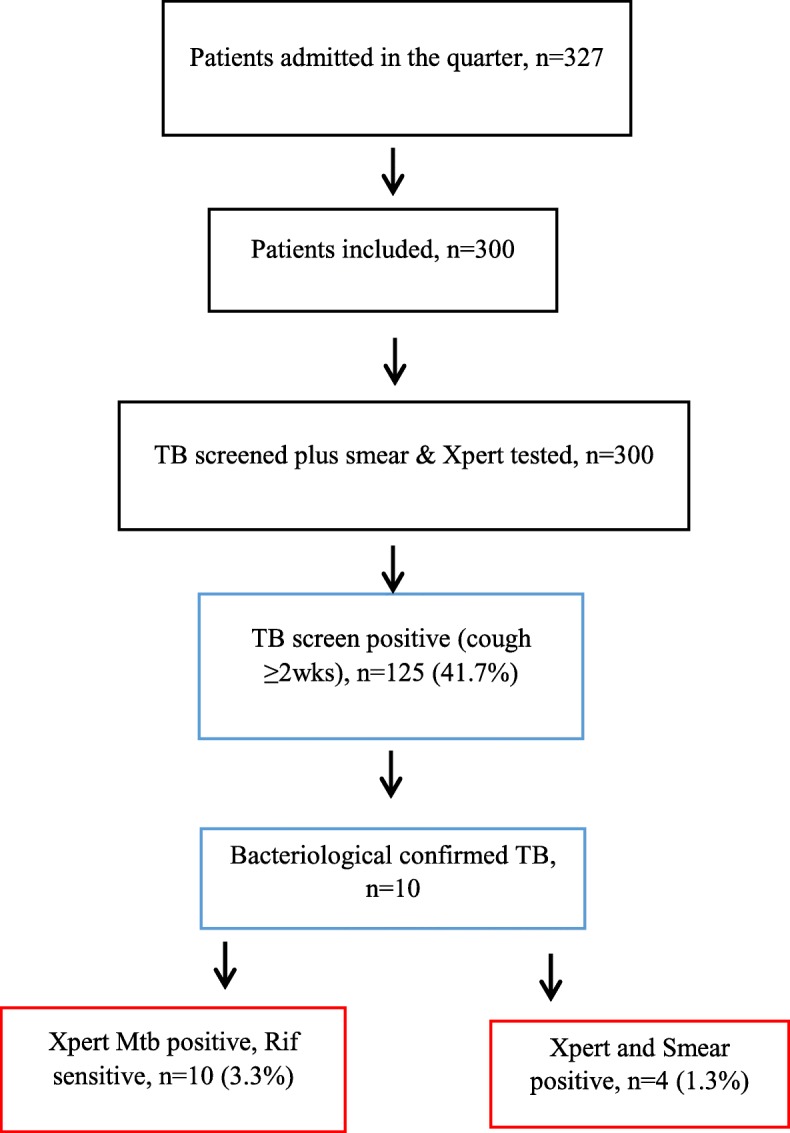
Flow diagram – TB screening and sputum result of patients in the medical wards

Of the 300 inpatients, 10 (3.3%) were found to be Xpert MTB positive / Rif sensitive and 4 (1.3%) were found to be both smear positive smear and Xpert MTB positive (Fig. 1). Anti-TB treatment was initiated for 9 out of 10 diagnosed TB patients - 1 patient died before their laboratory results became available. All patients with an Xpert MTB positive result reported a cough of 2 weeks or more, with in addition two or more other common TB symptoms reported. Severe pneumonia and HIV were the medical reasons for admission in 8 (80%) of the 10 detected TB cases (Table 3). The risk of TB disease among HIV positive patients was nearly 3 times higher (RR = 2.92) compared to HIV negative patients (Table 4).

**Table 3:** Patient characteristics, admission diagnosis and presence of TB symptoms amongst the 10 detected bacteriological confirmed TB cases

s/n	Age	Sex	BMI	HIV test	Admission diagnosis	TB symptoms	Smear result	Xpert result
1	22y	F	20.9	neg	CAP	Cough 1mth, fever, wt loss	neg	+ve
2	21y	F	20.0	U	Severe CAP	Cough 3wks, fever, night sweat	neg	+ve
3	26y	F	18.6	+ve	WHO Stage 4 RVI	Cough 2wks, fever, wt loss	neg	+ve
4	55y	M	21.0	U	Severe anemia	Cough 2wks, chest pain, wt loss	neg	+ve
5	22y	M	18.2	neg	IHD	Cough 2wks, fever	+ve	+ve
6	32y	M	20.3	U	Pneumonia	Cough 2wks, fever, wt loss	+ve	+ve
7	33y	M	20.5	+ve	WHO Stage 4 RVI	Cough 2wks, fever, wt loss, contact hx	+ve	+ve
8	23y	M	20.3	+ve	RVI	Cough 2wks, chest pain, fever, night sweat	+ve	+ve
9	31y	M	19.1	+ve	RVI	Cough 3wks, fever, night sweat	neg	+ve
10	41y	M	18.4	neg	Diabetes M	Cough 2wks, chest pain, fever, night sweat, wt loss	neg	+ve

*BMI* Body Mass Index, *neg* negative, *+ve* positive, *U* unknown, *CAP* Community Acquired Pneumonia, *RVI* Retroviral Illness (HIV/AIDS), *IHD* Ischemic Heart Disease, *wt* weight, *hx* history, *WHO* World Health Organization

**Table 4:** History of chronic illnesses (HIV, DM and chronic respiratory problems) and their association with bacteriological confirmed TB (*n* = 300)

Chronic illness	Bacteriological confirmed TB	Not bacteriological confirmed	Total	RR (95% CI)
HIV positive^a^	4	33	37	2.90 (0.86, 9.76)
Diabetes Mellitus	1	37	38	0.77 (0.09, 5.88)
Chronic respiratory problem	0	3	3	N/A
None of the above chronic illnesses	5	217	222	0.35 (0.10, 1.18)
Total	10	290	300	

^a^of the total patients with HIV test result recorded = 198

## Discussion

Major reasons why TB cases are being missed include not accessing care at all, accessing health services but not being diagnosed, or being diagnosed with TB but not being notified [12, 13]. In this study, over a 3-month period, we detected 10 undiagnosed TB cases among 300 patients admitted to the general medical wards of a large referral hospital in Ethiopia.

In both low and high TB burden countries, there is evidence that TB cases are missed by the health services, either due to misdiagnosis as something else or due to a lack of clinical suspicion [4, 5]. Missing TB cases has implications for both the individual and the community. Delaying treatment increases the period of infectiousness and thus the chance of transmission in both HFs and in the community. Furthermore, the severity of the disease worsens imposing higher medical costs for the patient and health system, with resultant poorer treatment outcomes [4]. South African and British hospitals implemented a ‘TB process-based performance tool’ as a novel method to evaluate accurate and timely diagnosis of TB disease, which helped also to assess the missed opportunities for TB diagnosis. It was found that simple clinical actions were omitted in many cases. For example, chest symptoms were not recorded for 39% of cases and sputum smear examination was not done in 85% of patients. Omission of basic history taking and request of sputum smear are common to nearly all settings [3]. Our study shows a similar picture - 125 (41.7%) of our study participants reported common symptoms of TB that should have prompted the HCWs to consider TB. However, these symptoms were not recorded and appropriate clinical actions did not follow, such as requesting a sputum test. As a result, TB cases were missed. That TB guidelines are not followed in a high burden TB country such as Ethiopia is of concern and will lead to missed opportunities in diagnosing TB cases. It would be important to see if the practices of the HCWs observed in our study are found in other large hospitals in the country.

The TB diagnostic landscape which for decades relied on smear microscopy, is now changing with the availability of new technologies such as the Xpert MTB/Rif assay and LED fluorescent microscopy. Of the 10 cases identified in our study, 6 were GeneXpert positive alone and 4 were both smear and GeneXpert positive. Both the WHO 2016 and National Ethiopian guidelines include GeneXpert as the primary test for the diagnosis of TB disease amongst people living with HIV [14, 15]. However in our study, 40% of the identified “missed” TB cases were HIV co-infected patients with a history of chronic cough, but who were not identified as presumptive TB cases and were not tested by GeneXpert.

The risk of TB transmission to patients and HCWs in the health care setting has been recognized for many years and adequate adherence to IC measures is key [7–9]. However, IC implementation has been reported to be inadequate in many HFs in high TB burden settings such as Ethiopia [16–18]. TB disease among HCWs is not routinely monitored in Ethiopia and measurement of nosocomial transmission is difficult since undiagnosed TB patients are an important source of transmission [10]. In this study, all the identified “missed” cases were symptomatic, and 40% being smear positive.

The fact that we purposefully selected just 1 big referral hospital in the country obviously limits the representativeness of our findings. However, the hospital selected is a major general tertiary referral hospital in Ethiopia and the finding necessitates immediate interventions to address the identified gaps such as poor practice of TB screening of inpatients, incomplete documentation, and lack of appropriate use of sputa testing. The findings of this study are preliminary and more evidence on missed TB cases in high workload health care settings in Ethiopia is urgently needed.

## Conclusion and recommendation

Our study suggests TB cases are being “missed” in the general medical inpatient wards in hospitals in Ethiopia. TB diagnosis requires that HCWs have a high degree of suspicion for TB disease and adhere to basic clinical practice and national TB guidelines. To avoid missing TB cases all inpatients especially those presenting with respiratory-related symptoms, should be systematically screened for TB disease as per the national guidelines. HCWs need to comply with basic clinical practice and processes i.e. full history taking, TB screening, clinical examination and appropriate investigation, to determine which persons seeking care need a diagnostic work up for TB disease. The use of the newer TB diagnostic technologies with higher sensitivity such as GeneXpert MTB Rif assay test should be better utilized by HCWs in order to enhance diagnostic yield amongst those patients identified as presumptive TB cases.

## Abbreviations

AFB: Acid Fast Bacilli
ART: Anti Retroviral Therapy
BMI: Body Mass Index
DOTS: Directly Observed Treatment, Short course strategy
FM-LED microscope: Fluorescent Light-Emitting Diode microscope
HCWs: Health Care Workers
HIV: Human Immuno-Deficiency Virus
IC: Infection Control
IQR: Interquartile Range
MDR-TB: MultiDrug-Resistant Tuberculosis
MTB: Mycobacterium tuberculosis
NSP: National Strategic Plan
SD: Standard Deviation
WHO: World Health Organization

## References

[1] World Health Organization 2014. Tb. Reach the 3 million. Find. Treat. Cure TB. Accessed, http://www.stoptb.org/assets/documents/resources/publications/acsm/WORLD_TB_DAY_BROCHURE_14March.pdf.

[2] World Health Organization 2015. Global Tuberculosis report 2015. WHO/HTM/TB/201522.

[3] Field N, Murray J, Wong ML (2011). Missed opportunities in TB diagnosis: a TB process-based performance review tool to evaluate and improve clinical care. BMC Public Health.

[4] Sendagire I, Schim Van der Loeff M, Mubiru M, Konde-Lule J, Cobelens F (2010). Long Delays and Missed Opportunities in Diagnosing Smear-Positive Pulmonary Tuberculosis in Kampala, Uganda: A Cross-Sectional Study. PLoS One.

[5] Greenaway C, Menzies D, Fanning A, et al. Delay in Diagnosis among Hospitalized Patients with Active Tuberculosis – Predictors and Outcomes. Am J Respir Crit Care Med Vol, 165. 2002:927–33. 10.1164/rccm.2107040. 10.1164/ajrccm.165.7.2107040 11934716

[6] Miller AC, Polgreen LA, Tuberculosis Unseen PPM (2015). Missed opportunities in diagnosis. Online Journal of Public Health Informatics.

[7] He GX, Van den Hof S, Van der Werf MJ (2010). Infection control and the burden of tuberculosis infection and disease in health care workers in China: a cross sectional survey. BMC Infect Dis.

[8] Corbett EL, Muzangwa J, Chaka K (2007). Nursing and community rates of Mycobacterium tuberculosis infection among students in Harare, Zimbabwe. Clin Infect Dis.

[9] Baussano I, Nunn P, Williams B (2011). Tuberculosis among health care workers. Emerg Infect Dis.

[10] Bantubani N, Kabera G, Connolly C (2014). High rates of potentially infectious Tuberculosis and multidrug-resistant Tuberculosis (MDR-TB) among hospitalized inpatients in KwaZulu Natal, South Africa indicate risk of nosocomial transmission. PLoS One.

[11] Federal Democratic Republic of Ethiopia Ministry of Health (FMOH). Revised strategic plan Tuberculosis, TB/HIV, MDR-TB, and leprosy Prev Control 2013, Addis Ababa, Ethiopia.

[12] David WD, Sanjay B, Jason RA (2013). Is passive diagnosis enough? The Impact of Subclinical Disease on Diagnostic Strategies for Tuberculosis. Am J Respir Crit Care Med.

[13] WHO/STB/TME. Assessment of the fraction of cases being missed by routine TB notification data, based on the "Onion" model. Microsoft PowerPoint - Onion model_AB.ppt. http://www.who.int/tb/advisory_bodies/impact_measurement_taskforce/meetings/ie_jul09_onion_model.pdf

[14] WHO 2016. Algorithms for managing people living with HIV who are suspected of having TB (ambulatory and seriously ill). www.who.int

[15] FMOH. Guideline for clinical and programmatic management of TB. Leprosy and TB/HIV in Ethiopia. 5th ed. Addis Ababa: April; 2012.

[16] Buregyeya E, Nuwaha F, Verver S (2013). Implementation of tuberculosis infection control in health facilities in Mukono and Wakiso districts, Uganda. BMC Infect Dis.

[17] Addis Ababa University. Assessment of knowledge, attitude and practice of TB infection control among medical laboratory professional in selected DOTS health facilities under Addis Ababa City Administration Health Bureau, Addis Ababa Ethiopia.

[18] Temesgen C, Demissie M (2011). Knowledge and practice of tuberculosis infection control among health professionals in Northwest Ethiopia.

